# Upcycling Apple and Carrot Pomace into Fortified Food Ingredients: Advancing Sustainability and Resource Efficiency

**DOI:** 10.3390/foods15091514

**Published:** 2026-04-27

**Authors:** Ramona Căpruciu, Simona Mariana Popescu

**Affiliations:** 1 Department of Horticulture and Food Science, University of Craiova, 13 A.I. Cuza Street, 200585 Craiova, Romania; ramona.capruciu@edu.ucv.ro; 2 Department of Biology and Environmental Engineering, University of Craiova, 13 A.I. Cuza Street, 200585 Craiova, Romania

**Keywords:** apple and carrot pomace, powder, composite foods, sustainability, circular economy

## Abstract

The generation of byproducts during the production of apple and carrot juices can negatively impact the environment. Using these byproducts as functional ingredients represents an integrated and innovative strategy for today’s food industry. This review aims to provide a comprehensive analysis of the progress made over the past decade in the applications of apple and carrot pomace in the food industry, with an emphasis on qualitative analyses (compositional, sensory, functional) in the context of promoting sustainability and resource efficiency. The main quality parameters of apple and carrot pomace and pomace powders, as well as of the composite food products in which they were incorporated, were examined with the optimal dosage. The analysis reveals the extent to which the studied powders can improve—or fail to improve—the nutritional, functional, or sensory parameters of composite products, while accounting for environmental impact and sustainable practices within the context of circular economy. It is found that the industrial reintegration of apple and carrot pomace has nutritional, functional, and bio-packaging development potential, with the main remaining challenge being the development of solutions to preserve the color and rheology of composite products.

## 1. Introduction

Food byproducts or food waste consist of edible or inedible fractions remaining after the production of various food products [1]. Although these two concepts are often used interchangeably, European legislation makes a clear distinction between them, defining food waste as discarded residues from food production with a high organic content, while food byproducts are residues from which high-value materials can be recovered and incorporated into various functional products [2]. The projected global population growth in the coming period will increase demand for food [3], accelerating the consumption of natural resources [4,5] and generating larger quantities of food byproducts that become food waste, thereby placing immense pressure on the environment [6]. Thus, according to FAO, 17% of global annual waste comes from byproducts of the juice and beverage industry, a share that could double by 2050 [7]. This proportion is also due to the fact that food waste occurs throughout the entire food chain, from production, storage, processing, distribution, and consumption to disposal [8], with 24% ending up in landfills and 22% being incinerated [9], which can negatively impact the environment.

The pomace resulting from industrial juice production finds its most valuable application in pharmaceutical and nutraceutical products, followed by the food and animal husbandry sectors, then in materials such as bioplastics and biopolymers, and finally in fertilizers, compost, or biofuel production. In this context, applications in pharmaceuticals, cosmetics, and food represent solutions for the effective utilization of food byproducts, with a positive environmental impact in line with the principles of the circular economy [10,11,12].

A current sustainable approach superior to the linear model of economy is the circular economy, which aims to improve conservation efficiency by recycling byproducts as valuable raw materials for new products, thereby reducing the use of natural resources and the generation of waste [13,14].

**Figure 1:**
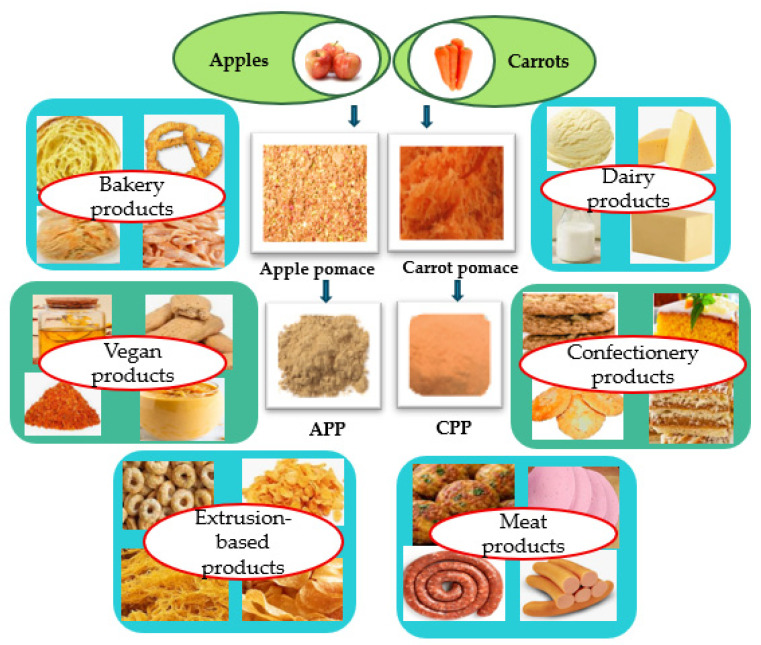
Applications of apple and carrot powders in the food industry.

The variety of ways in which fruit and vegetable byproducts re-enter the industry is significant, ranging from fortified ingredients and natural additives (colorants, preservatives, thickening agents [15]), 3D printing applications in the food industry [16], aquaculture feed (approximately 53 million tons of feed annually, according to [17]) to antimicrobial agents [18] or biorefining systems for holistic valorization [19], etc. Among these, the food sector offers significant potential to enhance the functionality of food products by incorporating fruit and vegetable byproducts in various forms, with powder being the most commonly used. The addition of pomace powders/flours in food products (bread, biscuits, meat products, dairy products, etc.) is a rational approach [20,21], based on a rigorous assessment of the risks and benefits regarding their use in the development of innovative food products within the context of the circular economy (Figure 1).

Fortifying certain foods with pomace powder derived from the industrial processing of apples or carrots offers nutritional and functional benefits, a fact confirmed by the impressive number of studies on this topic over the past decade (Section 4).

The recent importance attributed to these powders stems from their concentration of bioactive compounds, offering health benefits (antioxidants, anti-inflammatory agents, immunomodulators, etc.) [22,23,24,25,26].

Apple (*Malus domestica* L.) is one of the most widely consumed fruits in the world, used primarily for fresh consumption (70–75%) but also processed (25–30%) into products such as juice, cider, purees, jams, and dried products like chips or powder [27,28,29]. Among these products, nearly 75% of the fresh weight of apples is converted into juice, with the remainder discarded as pomace [30]. Apple pomace (AP), resulting from the extraction of juice, is a heterogeneous mixture consisting of peel and pulp residue (95%), seeds (2–3%), and stems (1%), and is one of the most extensively studied plant byproducts [31]. After pressing, due to its high moisture content, it is dried to inhibit microbial growth to a water content of 4.4–10% [32,33], and processed into powder for use in the food industry. The richness of apple pomace powder (APP) in substances with health-promoting effects (total phenolic compounds, soluble and insoluble dietary fiber, micronutrients, carbohydrates, etc.) [34,35] makes it a valuable sought-after ingredient for the fortification of certain foods.

Carrot (*Daucus carota* L.) is a root vegetable belonging to the Apiaceae family, with a global production of up to 42 million tons [36], with Asia being the main contributor to its production, followed by Europe [37]. It ranks among the most widely used vegetables in the food industry, with a high percentage of industrial processing, which leads to the generation of waste in the form of pomace. Carrot pomace (CP) is a rich source of minerals (potassium, iron, calcium, magnesium, zinc), vitamins (A, C, K, B), dietary fiber [38], carotenoids, particularly β-carotene, and other antioxidants such as lutein and zeaxanthin [15] that can be incorporated into various food products as fortifying ingredients [39,40].

The importance of apple and carrot pomace has been highlighted by research conducted over the past decade, which demonstrates that they are rich in valuable compounds that could be recycled in the food industry and related sectors (medical, pharmaceutical, livestock farming, aquaculture, etc.). Although the generation of these pomaces is continuous and inexpensive, most of it is still disposed of as waste. However, there is currently a research focus on fortifying certain food products with APP or CPP, with hopeful results in sectors such as baking, pastry products, confectionery, the dairy industry, the meat industry, etc. (Figure 1) as well as for replacing certain synthetic food additives such as food coloring, antioxidants, preservatives [41], and thickening agents [42].

In addition, the disposal of byproducts in landfills generates high levels of methane [43], hydrogen sulfide, and ammonia [44], while also providing ideal environments for the growth of pathogenic microorganisms and pests, serving as hotspots for the transmission of infectious diseases [45], and thus potentially endangering human health.

The selection of apple and carrot pomace for this study was based on their byproducts generated by the juice industry. Furthermore, their predominant use in sectors of the food industry, such as confectionery and baking, is relevant to the study of apple and carrot pomace, an aspect highlighted in Section 4. This study is significant for current food research because it highlights aspects of applied innovation resulting from the sustainable reintegration of apple pomace and carrot pomace, in powder form, into food composites that are, in terms of quality, superior to traditional products.

## 2. Methodology

This article conducted a comprehensive literature search across major scientific databases, including Web of Science, Scopus, ScienceDirect, PubMed, and Google Scholar, focused on the valorization of apple and carrot pomace. The search covered publications from the last ten years to capture both foundational and the most recent advances in the field. Keywords combinations were used to refine the search, such as apple pomace, carrot pomace, valorization, byproduct utilization, waste recovery, functional foods, composite foods, bioactive compounds, food applications, environmental impact, and LCA. The selected studies were based on the following inclusion criteria: peer-reviewed journal articles, reviews, and book chapters addressing chemical composition, bioactive compounds, processing techniques, functional applications, valorization pathways, environmental impact of waste, and articles published in English, with an initial result of 250 articles. Exclusion criteria included non-scientific reports, editorials, and conference abstracts without full text, duplicate publications, or studies with insufficient methodological detail, resulting in 200 articles for abstract screening to assess relevance to pomace valorization and full-text review for final eligibility. Out of these, 133 articles were identified as relevant to this review topic and were included in the final synthesis. Relevant data were systematically extracted from the selected studies to identify current knowledge on apple and carrot pomace valorization, supporting the identification of research trends, technological opportunities, and future directions with alimentary product development.

## 3. Qualitative Aspects of Apple Pomace (AP) and Carrot Pomace (CP)

**Figure 2:**
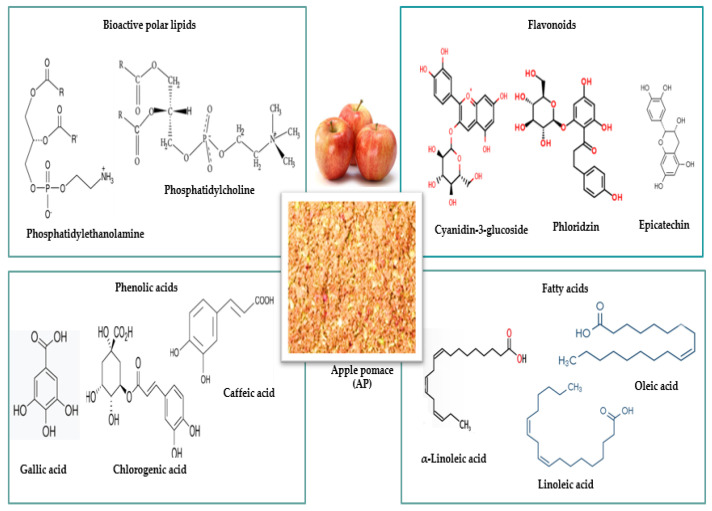
The main bioactive compounds in apple pomace.

**Figure 3:**
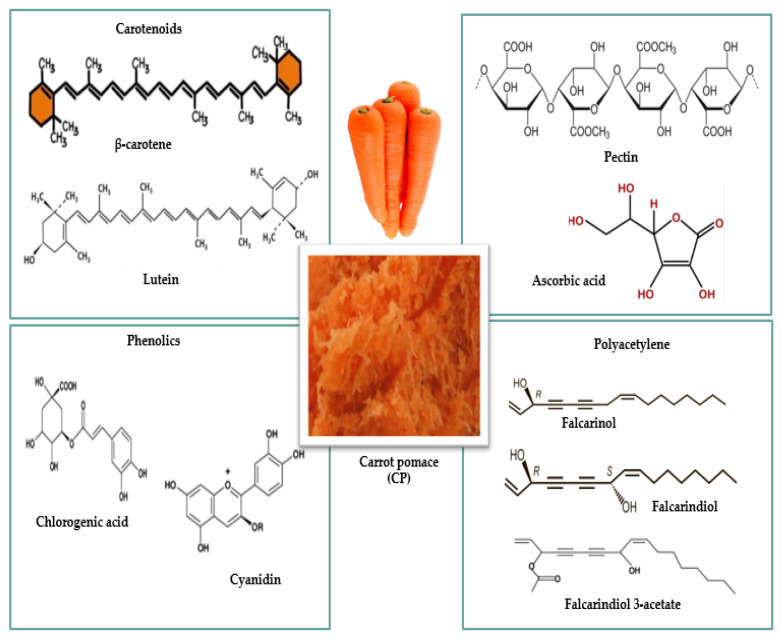
The main bioactive compounds in carrot pomace.

The main byproducts generated by the industrial processing of plant-based raw materials are pomaces, which can be used in the food industry as powders or flours (varying degrees of fineness/particle size), cakes, extracts, or tinctures. Some compounds in apple and carrot pomace have valuable nutritional (carbohydrates, proteins, lipids) and bioactive compounds properties (antioxidants, polyphenols, enzymes, dietary fiber, or trace elements) (Figure 2 and Figure 3), hence their recognized importance in innovative research in recent years (Table 1 and Table 2) for industrial applications in the food industry.

The reuse of these pomaces in the food industry can be achieved through techniques such as component separation (if necessary), drying, grinding (from flour with a known particle size to powder), purification (if required), encapsulation, and storage until use.

### 3.1. Quality of Apple Pomace (AP)

Over the past decade, AP has been used as an ingredient in food products due to its high content of bioactive compounds (Figure 2) and nutrients, which fluctuate with variety and processing type (Table 1).

In their study, Usman et al. [52] found that AP is rich in total phenolic compounds (9.75 mg/g) and dietary fiber (10.85%). AP is rich in total dietary fiber (30%), of which insoluble fiber and carbohydrates predominate [30]. Compared to insoluble fiber, soluble fiber has stronger physiological and bioactive properties, such as antioxidant activity, gelling capacity, viscosity, and fermentability [59,60,61]. Thus, the main components of insoluble fiber in AP are cellulose (6.7–40.4 g/100 g DM), lignin (14.1–18.9 g/100 g DM), and insoluble hemicellulose (approximately 16.4 g/100 g) [47], while the main soluble fiber in AP is pectin, accounting for up to 15% of the dry matter in pomace [62], gums, mucilages, and soluble hemicellulose, as well as other components with health-promoting properties. Another important aspect of dietary fiber in AP was studied by Antonic et al. [63], who found a significant increase in total fiber content in the fortified products, demonstrating that the fortification transfer is achieved, and can thus be declared functional. Furthermore, the analysis shows that fortification with apple pomace resulted in better texture, oxidative stability, and sensory parameters (particularly color) in animal-based food products compared to plant-based ones. The study by [46] showed that pectin can be used in the food industry as a thickening or gelling agent. Another method for increasing nutritional and functional value was investigated by Madrera et al. [27], who related that solid-state fermentation of apple pomace using indigenous cider yeasts leads to significant increases in phenolic compounds and nutritional composition (proteins by 23–49%, fats by 17–39%, and dietary fiber by 30–41%, compared to unfermented apple pomace). The possibility of using apple pomace to enhance the antioxidant activity of cider was studied by Bortolini et al. [64]. Their findings suggested that one way to recover the phenolic compounds remaining in the pomace is to add the pomace during cider must fermentation, thereby improving the sensory parameters, phenolic composition, and antioxidant activity of the cider.

Apple pomace has a low lipid content (below 5%), with a high percentage of unsaturated fatty acids [50]. Due to the physiological and bioactive properties of the soluble fibers in AP, food additives can be obtained for use in the food industry as stabilizers, thickening agents, emulsifiers, and fat replacers [59,60].

The polyphenolic components of apple pomace include major groups such as flavanols, such as (−)-epicatechin, (+)-catechin, and their polymers, such as procyanidins, glycosylated flavonoids of quercetin, hydroxycinnamates, dihydrochalcones (floretin 2-*O*-glucoside), hydroxycinnamic acids (dominated by caffeoylquinic acids such as p-coumaric acid, caffeic acid, and ferulic acid) and anthocyanins [28]. The proportion of flavanols in the pomace of apple varieties studied by Persic et al. [65] was significantly higher than in the fruit and juice, e.g., the pomace of the Majda variety (a local Slovenian cultivar), had the highest proportion of flavanols in the fruit (22%), juice (38%), and pomace (55%). These results indicate a high dependence of the fruit’s phenolic composition on variety, strongly correlated with the rate of enzymatic browning. The use of apple pomace as a source of phenolic compounds was also investigated by De Camargo et al. [66], who focused on the role of genetics in the biosynthesis and distribution of phenolic compounds, potential health benefits, and safety considerations. Cold-pressing apples results in the preservation of a higher phenolic content compared to conventional pressing [67], which could facilitate the extraction of bioactive compounds necessary for enhancing the functionality of food products. In their study, Antonic et al. [63] found that AP is a source of carbohydrates (14–17% starch) and micronutrients such as potassium, calcium, and magnesium, as well as vitamins such as vitamin C (22.4 mg/100 g dry weight) and vitamin E, with most of these compounds found in the seeds.

Regarding minerals, Gumul et al. [68] showed that Cu, Zn, and Mn, in particular, have the ability to stimulate antioxidant enzymes in the human body, improving their functionality and potentially eliminating the overproduction of free radicals in cases of autoimmune diseases. They also indicate an likely increase in total polyphenol content in gluten-free bread (1.02 mg gallic acid/100 g d.m., equivalent to 2 mg catechin/100 g d.m.), which is likely due to the Maillard reaction, as its products may react with the Folin–Ciocalteu reagent. The increase in phenolic acids in composite products may be due to yeast fermentation [69] and mechanical phenomena during dough mixing [70]. Another possible source of phenolic acids (particularly chlorogenic acid) may be the thermal degradation of quercetin derivatives, which are present in large quantities in AP, while the detection of lower levels of phenolic acids (particularly p-coumaric acid spermidines) compared to the control sample is due to the process of thermal decarboxylation [68]. The study on improving the technical and functional properties of apple pomace fibers through enzymatic treatment as a sustainable and effective approach to modifying the insoluble fraction was conducted by Díaz-Núñez et al., 2025 [71]. This research highlights the potential of optimizing enzymatic processing to transform fruit byproducts into new food ingredients with improved solubility. They identified the ideal conditions (temperature of 48 °C, hydrolysis time of 3.31 h at an enzyme concentration of 4.1 mL/kg DW) under which the solubility of AP increased by a factor of 1.70 compared to untreated AP, highlighting the potential of optimizing enzymatic processing to improve fiber solubility, thereby increasing the potential for using pomace as new food ingredients.

Therefore, apple pomace is notable for its rich composition of both nutrients and bioactive compounds, a characteristic that makes it suitable for reuse in various sectors such as pharmaceuticals, nutraceuticals, and food, with the ultimate goal of innovatively optimizing these resources to benefit human health.

### 3.2. Quality of Carrot Pomace (CP)

This vegetable is essential for human consumption and makes a significant contribution to human health [72] due to its high content of bioactive compounds, such as vitamins, carotenoids, anthocyanins, minerals, and dietary fiber [73,74].

In the food industry, during the processing of carrots for juice production, large quantities of byproducts are generated [75], with approximately 50% of the raw material consisting of pomace, which is then disposed in landfills or used as animal feed and manure [76].

Studies conducted over the past decade show that processing CP into powder and adding it in varying percentages to food formulations, due to its rich chemical composition (Figure 3), can result in fortified foods (Figure 1), with the applied technologies directly influencing the quality of the final product (Table 2).

The importance of carotenoids in CP in the diet stems from their role as natural pigments, but especially from their proven biological functions and effects [76]. In addition to its carotenoid, fiber, and ash content, ref. [54] found that CPP also contains vitamin C (1.53 mg/100 g), making it a suitable ingredient for functional food products. In a subsequent study, the findings of [56] showed that CPP has a high content of dietary fiber (43.59%) and carbohydrates. To preserve the bioactive compounds in the pomace, the use of appropriate pretreatment methods plays a crucial role. In 2026, Román et al. [77] enriched sunflower oil with lycopene extracted from carrot pomace without the use of chemical solvents, presenting a sustainable approach using the same improved infrared drying technology. In contrast, Richards et al. [78] found that freeze-drying of pomace is superior in terms of retained carotenoids, water holding capacity, and fat-binding capacity compared to dehydrated pomace, thereby enhancing the functional properties of carrot pomace. Other treatments such as the complex enzymatic method, ultrafine grinding, and high hydrostatic pressure were applied by Yu et al. [72] to CP, resulting in an increase in soluble dietary fiber content of up to 15.07%, with major health benefits (maximum cholesterol adsorption capacity, improvement in total antioxidant activity, etc.), the conclusion being that dietary fiber modified from CP using the three applied methods can be introduced as a functional ingredient in food production technology. Furthermore, Baskovtceva et al. [79] developed a technology utilizing enzymatic processing and impact-activated disintegration processing of CP (converted into CPP) to increase the percentage of beta-carotene in plant cells and demonstrate that this technology significantly increases the carotenoid yield in CPP by 17 times compared to the raw material.

Table 1 and Table 2 show significant variability in the chemical composition values for both AP and CP, which is influenced by factors such as apple variety, climatic, soil, and agrotechnical conditions, pomace production technology, and the specific conditions under which the pomace is obtained (environmental type, temperature, pH, and time), different extraction methods, the way results are expressed (for example, different types of phenolic compounds are used to calculate polyphenol levels), and, last but not least, particle size. For example, research shows that the extraction of bioactive compounds from food matrices via microwave-assisted extraction (MAE) is more efficient (faster and cheaper) compared to conventional methods (Soxhlet and heated reflux extraction), due to the fact that it generates heat that causes molecules or ions to move from the interior to the exterior simultaneously with the evaporation of moisture from the biomass [80]. The ultrasonic-assisted extraction (UAE) technique utilizes the acoustic energy generated by ultrasonic waves (cavitation process) that cause the compression and expansion of the food matrix, increasing the permeability of the cell wall, thereby improving the extraction of bioactive compounds. A higher amplitude increases the compression and refraction cycle [81].

The results of the study conducted by Zhang et al. [82] showed that particle size influences the extraction of bioactive compounds. In their study, they used two apple pomace samples (AP1, 500–750 μm, and AP2, 100–250 μm) and demonstrated that a greater amount of bioactive compounds is extracted from smaller particles (AP2, 2.81 ± 0.29 mg GAE/g, and from AP1 it was 2.23 ± 0.03 mg GAE/g). The nutritional and bioactive composition of apple and carrot pomace enables the innovative development of composite food products by applying methods and technologies that add value to current production processes, with a focus on enhancing sustainability.

## 4. Applications of APP and CPP in the Food Industry

The relevance of research in this field arises from practical innovations in the development of functional foods that positively impact consumer health [83,84]. This is complemented by the low cost of raw materials and the positive environmental impact. Given the growing interest in nutrient-rich foods and bioactive compounds with health benefits (Figure 2 and Figure 3), the conversion of AP to APP and CP to CPP offers real potential as ingredients in the production of various functional food products.

Qualitative analysis of pomace powders generated during the industrial processing of apples and carrots reveals a wealth of valuable compounds (dietary fiber, polyphenols, micronutrients) that can be reintroduced into the food cycle to create nutritious foods with functional properties. Research on these pomaces is extensive, yielding impressive scientific data that meet the demands of consumers concerned with a healthy lifestyle, as well as those who primarily consume plant-based foods. Currently, the food market offers a wide range of products fortified with APP (Table 3) and CPP (Table 4), but the challenge is to maintain the sensory properties of standard, unmodified products.

### 4.1. Applications of the APP in the Food Industry

Over the past decade, there has been an increase in the use of APP-based composites across various food sectors, ranging from finished bakery and confectionery products, meat products, and oils to food additives—such as texturising and coloring agents—and biodegradable packaging, with the optimal percentage typically being 10% (Table 3).

The effect of replacing wheat flour with 25%, 50%, and 75% with fine and coarse APP was studied by Zlatanović et al. [85] in the context of industrial-scale biscuit production, evaluating sensory properties, the content and retention of dietary compounds, and antioxidant activity at the time of production and one year after storage. The results showed that coarse apple pomace flour improved the evaluated parameters, with biscuits containing an optimal amount of coarse apple pomace flour (50%) recording values several times higher than those of the control biscuits, including sensory parameters such as intense fruity aroma and crisp texture. Additionally, Kırbaş et al. [88] analyzed the dough rheology and physicochemical characteristics of gluten-free rice flour-based cakes enriched with APP, CPP, and orange pomace powder (OPP) (0, 5, 10, and 15%) as fiber sources, examining the flow behavior and viscoelastic parameters of the doughs enriched with dietary fiber, with positive results. It was found that the addition of pomace powders increased the specific weight of the dough and the hardness of the crumb, and decreased the specific volume of the cakes.

An innovative study by Carpes et al. [91] evaluated the volatile profile of a biodegradable film made from cassava starch extruded with varying percentages of APP and found that the film containing 8% apple pomace had a higher total phenolic content and antioxidant activity compared to the control film, the general conclusion being that apple pomace could be used as a functional ingredient in biodegradable packaging for the food industry, as it also possesses antibacterial properties superior to other films.

During the extrusion process, thermal and mechanical stress act together on the dietary fibers in food byproducts in a non-uniform manner, hence the difficulty in accurately measuring or calculating their structure and functional properties. In this regard, Schmid et al. [103] applied defined thermal and/or mechanical stress to APP and found that as temperature (and mechanical stress) increased, the soluble dietary fiber content rose, while the insoluble fiber content decreased, with greater sensitivity to high temperature than to mechanical stress.

In recent years, efforts have been made to use APP as a dietary fiber supplement in bread-making. In the study conducted by Gumul et al. [89], wheat dough was fortified by adding apple pomace at varying percentages (10, 20, 30, and 50%), with each product being qualitatively analyzed (total polyphenol content, individual polyphenols, dietary fiber, and physicochemical composition). Additionally, hardness and cutting resistance decreased relative to the control samples, while water absorption capacity remained stable, except for pasta with a 50% apple pomace addition. The aim of the study conducted by Mandache et al. [86] was to analyze the influence of using apple, cherry, and peach power pomace on the content of bioactive compounds and antioxidant activity in bakery products enriched with different proportions of pomace (5%, 10%, and 15%), observing a progressive increase in the content of bioactive compounds and antioxidant activity, with synergistic potential among all components, depending on the percentage of pomace added.

APP dried using different methods was ground into powder and used to replace 20% of the wheat flour in biscuit formulation [87]. They examined the overall composition, with differences observed mainly in fiber content (9.82–11.75%). The conclusion was that the drying method (biscuits with added freeze-dried pomace are lighter) and temperature influence both the physicochemical properties of the APP and those of the resulting biscuits (reduced red hues and increased yellow tones, increased firmness correlated with increased cutting force—30% higher), resulting in higher fiber content (9.82–11.75%).

Dried and ground to powder, apple pomace also finds application in the meat industry [41], being added to the recipe for Italian salami at proportions of 7% and 14% and subjected to a 25-day curing process. The resulting products underwent physicochemical, microbiological, and colorimetric analyses at well-defined intervals (every 5 days), with nutritional and sensory evaluations conducted upon completion of the maturation process. A slightly higher overall acceptability was observed for the 7% apple pomace addition, with the resulting product enriched with fiber and phenolic compounds, which correlated with a lower fat and calorie content. The results confirm the functional value added by AP to classic Italian salami.

An innovative mayonnaise formula with added value from apple pomace powder was developed and studied by Mangiapelo et al. [90], resulting in improved microbiological, chemical, sensory, and rheological parameters.

The production of an APP-based hydrogel was studied by Cossignani et al. [42]. They subjected the APP to different conditions: drying in an oven at 55 °C for 12 h; homogenization and drying in an oven at 55 °C for 12 h; homogenization and freeze-drying; grinding and sieving, yielding three particle sizes (>400 µm, 250–400 µm, and <250 µm). The hydroalcoholic extracts of these fractions, obtained via ultrasound-assisted extraction, were analyzed to compare total phenolic content (TPC), antioxidant properties, and phenolic profile. Higher values of total phenolic content and antioxidant capacity were recorded in the hydroalcoholic fractions comprising AP (250–400 μm) and AP (>400 μm), with oven-dried AP exhibiting both antioxidant activity and thickening capacity, specifically for use in food packaging.

Thus, based on the sensory and compositional characteristics, it can be concluded that food products fortified with apple pomace may exhibit improved nutritional, bioactive, and technological properties.

### 4.2. Applications of CPP in the Food Industry

CPP is versatile in its applications, offering physicochemical, nutritional, and functional improvements to composite foods, in most applications, the optimal incorporation rate being 10% (Table 4).

Carrot juice processing yields 30–50% pomace, only a small percentage of which is put to good use (as a functional ingredient, animal feed, or fertilizer), with the majority becoming waste that causes environmental problems. Recent studies demonstrated the potential of CPP to enhance the functionality of foods such as sausages, bread, milk and dairy products, and baked goods such as cookies, cakes, and gluten-free products, etc. (Figure 1, Table 4).

In this context, Yadav et al. [92] evaluated the texture, color, sensory, physicochemical, and nutritional characteristics of chicken sausages to which wheat bran and dried CP were added. The results showed that a 6% of each proposed ingredient resulted in products with very good acceptability, increased dietary fiber content, and the ability to be stored at refrigeration temperature for up to 15 days. By adding CPP and substituting carrot juice concentrate for water in the dough of certain pastries, ref. [104] demonstrated significant sensory improvements. An increase in moisture, firmness, color difference, and browning index was observed, while specific volume decreased, with consistency and elasticity remaining unaffected.

An evaluation of biscuits conducted by Sahni et al. [95] showed that the use of CPP increases the weight, thickness, and hardness of the biscuits, while the aspect ratio, expansion factor, and diameter decrease as the proportion of CPP in the flour mixture increases (10% yields the best characteristics). An increase in crude fiber, ash, and moisture content was observed, along with a decrease in carbohydrate and protein content; fat content did not show significant variations. From a sensory perspective, color, taste, and aroma improved, while the textural score decreased as the percentage of CPP in the composite increased.

The study on composite pastas (10, 20, and 30% CPP, beet and APP) conducted by Kultys et al. [58] highlighted that a 10% addition improves technological properties (higher dietary fiber content, water absorption, swelling index). Furthermore, the resulting pasta received positive ratings in consumer acceptance tests, offering an optimistic outlook for the future of this type of product. The conclusion is that using pomace (carrot, apple, and beetroot) in pasta production increases nutritional value while reducing food waste.

The use of colorimetric tests by Ziobro et al. [93] aimed to verify the retention of antioxidants present in bread made from wheat flour enriched with CP. The study found that adding 15% CPP significantly affected the dough’s technological and sensory properties (intense carrot aroma and persistent orange color). The study showed that the antioxidant properties of the CPP are largely preserved during baking, thereby increasing the antioxidant potential of wheat flour bread by 32%. This may support the recommendation to use CP in the production of breadcrumbs for coating and for incorporation into products that withstand high processing temperatures. Several types of composite bread (5%, 10%, 15%, or 20% CPP) were qualitatively evaluated by Begum et al. [54] using microbiological, physicochemical, and sensory methods. The study found that adding CPP increases the nutritional and sensory value of composite bread, while reducing specific volume and elasticity. A 10% addition resulted in the product with the highest overall acceptability, superior physical properties, and nutritional quality. These findings validate the use of CPP as a raw material or natural colorant to improve the nutritional and sensory value of composite bread, and it can also be utilized in the production of other types of food. In their research Ghadimi et al. [94], investigated the effect of CPP at different concentrations (10%, 20%, and 30%) on physicochemical properties (specific volume, moisture content, and fiber content increased, while fat and protein content in the final product decreases), sensory properties (color parameters, such as redness and yellowness, increased, while hardness and brightness decreased), and rheological properties of the bread (porosity increases).

Tests on the characteristics of the cookies were made by Andrejko et al. [97], who determined the characteristics of oatmeal cookies with added APP (5%, 10%) and CPP (5%, 10%), finding reduced hardness and increased elasticity and consistency. The use of these pomaces led to a decrease in the color brightness parameter, and in the case of biscuits enriched with CP, a chromatic shift toward red and yellow was observed. Furthermore, the increase in nutritional quality was significant (compared to the control sample) due to the addition of polyphenols, fats, and minerals (P, K, Ca), although a lower protein content was recorded. The study results indicate the importance of incorporating apple and carrot pomace for improving the sensory and nutritional parameters of oat cookies.

The enhancement of the dough quality of gluten-free cookies with different levels of CPP (0, 10, 20, and 30%) in a hydrocolloid (HC) mixture was investigated by Majzoobi et al. [98]. They demonstrated that the introduction of CPP + HC leads to improved physical parameters: increased dough viscosity compared to the control sample, decreased density and pH with the addition of CPP, a softer, more elastic, and easier-to-chew texture, and an improved uniformity index (the cake with 30% CCP + HC showed increased uniformity). The addition of CPP intensified the color of the cakes (darker compared to the control sample), while the inclusion of HC had no effect on the appearance and color of the cakes. It was concluded that the inclusion of 30% CCP and 20% CPP + HC improved the physical and sensory characteristics of gluten-free cookies.

The evaluation of gluten-free corn flour cookies enriched with carrot powder and stevia, conducted by Ibrahim et al. [105], showed that, from a sensory perspective (shape, aroma, color, texture), the enriched cookies significantly outperformed the control cookies. The enriched biscuits demonstrated improved physical characteristics (greater thickness and diameter, with a controlled spread ratio). Fortification led to significant improvements in protein, fiber, and mineral content, while simultaneously resulting in a lower total carbohydrate content compared to the control biscuits. Other recent studies aimed to develop and characterize gluten-free products by adding CPP. Among these, the gluten-free pasta whose sensory and nutritional characteristics were studied by Florença et al. [101] (composite pasta samples based on wheat and buckwheat flours with 5% and 10% carrot powder added), and the gluten-free muffins were formed by Mankutė et al. [102], incorporating CPP. The pasta samples were analyzed physically and sensorily, with positive results regarding hydration, cohesion, and cooking properties. Although brightness and color parameters (redness and yellowness) increased with the addition of carrot powder to the mixture, higher gelation temperatures and maximum viscosities, as well as darker color shades, were recorded compared to the control samples. The addition of CPP led to an increase in nutritional parameters (carotenoids and phenolic compounds). For the gluten-free muffins, six samples were prepared by partially replacing corn flour with CPP (5–10%) and xanthan gum (XG). The results showed that XG significantly reduced hardness compared to the control, while the effect of adding CPP on texture was concentration-dependent: (5% reduces hardness, 10% AP does not improve softness). The combined use of CPP and XG improved structural properties and increased dietary fiber content.

A study to develop a process for producing a value-added carp roe salad by incorporating CPP was conducted by Rațu et al. [100]. The process involved using CPP at 6% and 12% and evaluating the impact of these levels on the physicochemical and sensory properties of the roe salad, a composite product. An enrichment in carotenoids and, at the same time, an increase in antioxidant activity (550.66 ± 9.25–588.32 ± 9.41 μM TE/g DW) of the developed salad were observed. The color and texture profile improved, with the 12% addition being best perceived in terms of flavor and color. The enrichment of sunflower oil with valuable substances extracted from CP was analyzed by [37]. The use of modern Box–Behnken design technology for carotenoid extraction by MW-HPCO_2_ (microwave-assisted high-pressure CO_2_ system, in which temperature and time factors—significantly shorter compared to other techniques—significantly influenced extraction efficiency). Analysis of the chemical properties of the enriched oil demonstrated its oxidative stability, a factor that makes the MW-HPCO_2_ system considered safe, efficient, and environmentally friendly for applications in the food or pharmaceutical industry.

Increasing dietary fiber intake by incorporating CPP (2, 4, and 6% (*w/w*)) into the production of milk-based beverages as a dietary fiber source was studied by Rezvani et al. [99]. The results showed changes in color, an increase in apparent viscosity and solids content, and a slight increase in acidity (from 0.17 to 0.19%), using pectin with a high methoxyl content (0, 0.05, 0.1, and 0.15% *w/w*) as a stabilizer. The results showed that adding 0.1% pectin stabilizes the milk drink containing 4% CPP and reduces particle sedimentation, resulting in a beverage rich in dietary fiber, carotenoids, and vitamins. The evaluation of beef meatballs with added CPP in different proportions (1.0%, 3.0%, and 4.2%) was conducted by Richards et al. [74]. The product was analyzed for composition, and physical parameters such as water holding capacity (WHC), cooking yield, and texture, as well as sensory parameters. The study’s findings suggest that incorporating up to 3% CPP into beef meatballs improves the given functional properties and increases dietary fiber content while maintaining sensory quality.

Beyond their functional importance, these studies offer a practical and sustainable approach to utilizing carrot byproducts. These sustainable approaches utilize CPP in composites to produce foods fortified with antioxidants, pigments, fiber, minerals, and proteins, possessing bioactive, nutritional, and sensory properties that are increasingly recognized and valued by consumers.

The compositional richness of the studied pomaces, detailed in Table 1 and Table 2, determines their interaction with the food matrix once incorporated into a food composite (Table 2 and Table 3). One of the most well-known interactions is the increase in antioxidant capacity due to the presence of bioactive compounds [93] (e.g., polyphenols protect lipids from oxidation during storage [83]). The high fiber content (with pectins playing a crucial role) leads to an increase in water holding capacity (WHC), thereby influencing viscosity—a key characteristic in various food sectors such as dairy, meat [41], confectionery [86], or vegan products (Figure 1). Furthermore, this results in higher yields for hydrated products. The increase in oil holding capacity (OHC) is an aspect that enables the stabilization of food emulsions [90] where pomaces have been added in various percentages (Table 3 and Table 4).

Thus, the integration of APP and CPP into food composites modifies technological parameters such as rheology; for instance, increasing the pomace powder content (according to the data recorded in Table 3 and Table 4) increases dough consistency while degrading the gluten network, which impacts the elasticity and volume of the final product [89,93,94]. Since the studied pomaces provide fruity and acidic notes (APP) or sweet notes (CPP), they can be utilized to enhance the aromatic profile of the finished product or to mask bland flavors in certain food matrices [106]. Additionally, processing stability is improved (e.g., during baking, pectin acts as a hydrocoloid, enhancing gas retention and volume depending on the percentage of added pomace). Therefore, reintegrating these byproducts into the food industry facilitates the development of innovative solutions grounded in resource sustainability.

## 5. Environmental Considerations

Industrial processing contributes significantly to the generation of fruit and vegetable waste [58], with 25–30% of byproducts remaining after processing [107,108]. For example, ref. [109] found that 54.4% of fruit and vegetable byproducts came from juice processing, 43.98% from fruit salads, and 37.06% from vacuum-packed fresh-cut vegetables, making fruits the raw materials that generate the largest quantities, with apples being the subject of several studies in this regard ). Fruits and vegetables produce larger quantities of pomace that become waste due to their high water content, which makes them highly perishable and accelerates the degradation process, along with other factors such as microbial spoilage, physiological disorders, and mechanical damage [110]. The study by Lyu et al. [30] showed that the CP resulting from juice extraction is usually discarded as waste, which can lead to environmental problems and even endanger public health, although it can be used directly or after minimal processing in the functional processing of certain foods due to its content of valuable nutritional (carbohydrates and minerals) and bioactive (phenolic compounds, dietary fiber) compounds.

Most of the CP [74] and organic pomace in general ends up in landfills [111] where, due to a higher decomposition rate compared to other types of materials sent to landfills [112], large amounts of methane are produced—a gas with a higher global warming potential than CO_2_ [43,113]. Other gases released during the decomposition of food waste include hydrogen sulfide and ammonia, which contribute to pollution and the emergence of unpleasant odors [44]. Furthermore, landfills are ideal breeding grounds for pathogenic microorganisms and pests that act as vectors for transmitting various infectious diseases, posing a threat to human health [45].

One method for quantifying the environmental impact of the AP and CP resulting from the juice production process is to calculate the carbon footprint of a product or process through life cycle assessment (LCA) [114]. Life cycle assessment (LCA) estimates the environmental impact over the entire life cycle of a product [115], from raw material extraction through production, processing, distribution, consumption, and the generation of byproducts and, consequently, waste [116]. Environmental impact has been calculated in terms of global warming potential, in the study conducted by Scherhaufer et al. [117], which demonstrated the acidification and eutrophication potential of nine staple food products, utilizing over 134 existing LCA studies at the European level, with apples being the first mentioned. In terms of climate change impact, the LCA study of [118] on fresh unpacked carrots and frozen carrots revealed that the latter had a higher contribution to climate change with 0.614 kg CO_2_ eq compared to 0.186 kg CO_2_ eq for fresh carrots. For fresh carrots, the highest impact was attributed to transportation (46–50%), whereas highest impact was attributed to post-harvest handling for frozen carrots (42%). The combination of LCA with cleaner production principles (e.g., soil analysis for fertilizer recommendation, irrigation based on water balance, technical recommendations for the use of pesticides) for carrot farming examined by [119] lead to a reduction of greenhouse gas emissions from 0.12 kg to 0.07 kg CO_2_ eq/kg of the produced carrot. The LCA analysis conducted by [120] for conventional and organic carrot production showed that water footprint for organic production (WF = 1.9 m^3^ha^−1^) was five times lower than conventional production (WF = 10.4 m^3^ ha^−1^), with the highest impact for water footprint shaping the individual environmental impact categories studied being fertilization (67.0–67.7%) and carrot harvesting (41.9–43.1%) in the case of organic production.

The valorization of AP and CP through the recovery of bioactive components, such as anthocyanins, flavonoids, phenols, fiber, carotenoids, proteins, essential oils, micronutrients, and pigments, along with their incorporation and transformation into functional products [45,121] (research in Section 3.1 and Section 3.2) reduces the amount of waste. This has implications for the transition toward circular economy and a more sustainable environment [122] by reintroducing byproducts such as pomace into the food chain [84,123]. One way to utilize bioactive compounds from AP and CP is highlighted by Kumar et al. [107], who showed that in the food industry they find application in the development of edible films for the production of value-added foods, an aspect that represents an innovation in their sustainable use. In the search for solutions to minimize the environmental impact of plant waste, ref. [124] found that pomace rich in polysaccharides and proteins (including apple and carrot pomace in the study) can be used in the development of eco-friendly bioplastics or biocomposites with low environmental impact as sustainable alternatives to conventional plastic packaging used for food. Furthermore, the study highlights that through processes such as hydrolysis or digestion, innovative products can be obtained that adhere to the principles of circular economy, with a significant impact on food packaging in the near future and potential applications in food protection and extending shelf life. In their research, ref. [125] demonstrated that pyrolysis, hydrothermal carbonization, and physicochemical activation processes applied to fruit pomace lead to the production of a greater quantity of biochar, providing theoretical and experimental insights, thereby contributing to the reduction of waste from fruit pomace. Biochar from apple pomace was successfully developed by [126] in accordance with green chemistry principles. Carrot waste was used for the preparation of biochar by Elkhalifa et al. [127] as a single component and blended with cucumber, and tomato for hydrogen generation, with the mixture of the tree components having the highest yield. In the study of Molinuevo-Salces et al. [128], bioethanol concentrations of 50 g L^−1^ were obtained from apple pomace. The inclusion of apple pomace as feed in dairy cow diets examined by Xue et al. [129] revealed a reduction of CO_2_ equivalent emissions by 13%, with reductions of enteric CH_4_ production by 6.3% and CH_4_/feed intake by 10.6%.

Fruit and vegetable wastes—such as carrot and apple pomace—directed toward valorization pathways like animal feed, biochar, and biofuel production represent terminal uses. They do not preserve the nutritional value of the biomass [130,131] compared to the extraction of functional compounds that can be used for value-added products in the food industry.

## 6. Limitations

However, several factors influence the valorization of AP and CP, the most important being their differing compositions and characteristics, inconsistent extraction techniques (Table 1 and Table 2), and the lack of universally accepted methods that adhere to the principles of green chemistry [43]. Another factor is the presence of impurities, pesticide residues, and other chemicals in fruits and vegetables, which can contaminate byproducts intended for recovery and reduce their purity and quality [132]. Therefore, optimizing extraction methods and implementing specific guidelines and regulations are necessary for the valorization of fruit and vegetable byproducts to obtain finished products safe for consumption [45].

The color change of the composite product is another factor limiting the addition of large amounts of APP or CPP. In this regard, studies indicate that polyphenols often undergo enzymatic browning or thermal degradation (Maillard reaction), resulting in a darker color of the products [68]. Furthermore, the entire process of valorizing byproducts by transforming them into functional products must be scalable in production and cost-effective. In this context, Konjevod et al. [133] analyzed the potential of methodologies for valorizing waste from fruit and vegetable pomace (including CP), starting with mixed fermentation processes in kombucha production, with economic viability based on establishing partnerships between different production units that allow one unit to benefit from the byproducts generated by another unit. The ultimate goal is the successful implementation of a zero-waste strategy.

## 7. Conclusions

Apple and carrot pomace exhibit significant potential as primary or secondary ingredients in food applications, enhancing composite quality while adhering to rigorous food quality and safety standards.

The innovative reintegration of apple and carrot pomace into various food matrices increases their nutritional and functional value and mitigates environmental impact by reducing waste accumulation. This approach promotes resource efficiency within the frameworks of sustainability and the circular economy.

The foremost challenge for current and future food research remains the preservation of sensory parameters—specifically color—in pomace-based composite products, alongside developing solutions to optimize rheological properties while maintaining key nutritional profiles.

## Figures and Tables

**Table 1:** Chemical composition of apple pomace–AP.

Chemical Composition (%) (DW)		Detection Technique **	References ***
Dietary fiber	35.8 insoluble; 20.1 soluble.	The enzymatic–gravimetric method	[27]
	33.5 (total pectin)	Electric heavy-duty extractor (Turmix de Mexico S.A. de C.V, Queretaro, Mexico)	[46]
	5.3–12.4	by a theoretical calculation, considering the quantity added to the sample	[47]
	5.3	The enzymatic–gravimetric method	[48]
	26.5	AOAC Official Method 991.43 using the Megazyme kit K-TDFR-100A (Cedarlane Laboratories Ltd., Burlington, ON, Canada)	[33]
	42.6 total fiber of which 8.2 is soluble and 32.9 is insoluble	AOAC methods (labeled 991.42, 993.19 and 985.29)	[49]
	39.5 (total fiber)	Gravimetrically after hot water extraction, followed by precipitation with acidified ethanol	[50]
Total polyphenolics * (mg GAE 100 g^−1^ DW)	between 2.2 and 4.6	The photometric method using Folin–Ciocalteu reagent	[49]
	652.7 (Auksis cultivar) 472.5 (Alesja cultivar) 716.1 (Antej cultivar)	The photometric method using Folin–Ciocalteu reagent	[51]
	45.1	Soxhlet extractor	[48]
	312.3	The Folin–Ciocalteu colorimetric assay	[50]
Carbohydrates	4.7	HPLC and NIR	[27]
	7.4 (total sugars)	HPLC with a refractive index detector	[48]
	19.8	The differences in mean values, 100-(sum of percentages of ash, moisture, protein, and lipid)	[47]
	45.1	The Megazyme kit K-SUFRG (Cedarlane Laboratories Ltd., Burlington, ON, Canada)	[33]
	37.3 (reducing sugars)	The dinitrosalicylic (DNS) acid method	[50]
Total protein	3.5	The Kjeldahl method	[27]
	3.8	AACC (2000)-30.01 (FP-528 Nitrogen/protein Determinator, LECO Corporation, St Joseph, MI, USA)	[33]
	4.8	spectrophotometer (xMark™, Bio Rad, Hercules, CA, USA)	[46]
	1.9	The Kjeldahl method	[48]
	3.4	The Kjeldahl method	[50]
Total fat	1.8	The Soxhlet extraction	[27]
	3.8	SGS Canada Inc. (Burnaby, BC, Canada)	[33]
	0.6	AOAC (2007)	[46]
	1.7	AOAC (1997)	[47]
	0.8	The Soxhlet extraction	[48]
	4.4	The Svennerholm solvent extraction method	[50]
Moisture	15.3 to 21.6	According to the Standard ISO 6496:1999	[51]
	8.5	AOAC (2007)	[46]
	8.9	AACC 2000 standard method (method no. 44-15A)	[52]
Ash	6.3	AOAC (1997)	[47]
	3.8	The gravimetric method (AACC Method 08-01)	[48]
	1.5	Ash was measured as the weight lost after ashing at 600 °C for 2 h.	[50]

* (mg GAE/100 g DW): mg equivalent of gallic acid per 100 g of dry weight; ** AOAC—Association of Official Analytical Chemists; DNS—dinitrosalicylic acid method; HPLC—High-Performance Liquid Chromatography; *** the last 10 years.

**Table 2:** Chemical composition of carrot pomace—CP.

Chemical Composition (%) (DW)		Detection Technique *	References **
Dietary fiber	20.1–33.3	Megazyme kit K-TDFR-200a 04/17 (Megazyme Ltd., Bray, County Wicklow, Ireland)	[53]
	13.1	AOAC (2010)	[54]
	25.6–31.8	The Foss method (Fibertec™ 2010 Automated Crude & Detergent Fiber Solution, Slangerupgade DK-3400, Hilleroed, Denmark) according to AOAC 978.10 procedure	[55]
	43.6	AOAC (2005). 18th Edition, Association of Official Analytical Chemists, Washington DC, Methods 935.14 and 992.24	[56]
β-caroten	11.8	AOAC, 1980 (UV/V spectrophotometer)	[54]
	14.9	Ultrasound-assisted extraction: RSM with central-composite design	[57]
	48.3 (total carotenoids)	Box–Behnken design for carotenoid extraction by MW-HPCO_2_ (sistem de CO_2_ de înaltă presiune asistat de microunde)	[40]
Carbohydrates	46.5–58.9	The differences in mean values, 100-(sum of percentages of ash, moisture, protein, and lipid)	[53]
	49.2–53.1	The differences in mean values, 100-(sum of percentages of ash, moisture, protein, and lipid)	[55]
	52.7	LC-HRMS	[56]
Total protein	6.9–9.1	The ICC method (105/2)	[53]
	6.3–7.3	The Kjeldahl method	[55]
	5.83	AOAC (2005), Methods 935.14 and 992.24.	[56]
Moisture	30.0	AOAC (2005), Methods 935.14 and 992.24.	[58]
	16.6	AOAC (2005) Methods 935.14 and 992.24.	[56]
Ash	5.3–5.9	ICC (105/1)	[53]
	6,7	AOAC, 2010	[54]
	5.4–6.9	AOAC (2006) Method 925.36	[55]
	6.1	AOAC (2005) Methods 935.14 and 992.24.	[56]

* AOAC—Association of Official Analytical Chemists; ICC—International Association for Cereal Chemistry; LC-HRMS—high-resolution liquid chromatography–mass spectrometry analysis, RSM—response surface methodology. ** the last 10 years.

**Table 3:** The effect of adding APP to certain food composites.

Food Composites	Application Conditions	Results	References
cookies	25, 50 and 75% (fine and coarse)	↑ sensory parameters (flavor and texture) ↑ antioxidant activity (50% optimal value)	[85]
	5, 10 and 15%	↑ bioactive compounds ↑ antioxidant activity (10% optimal value)	[86]
	20%	↑ dietary fiber content ↓ color ↓ increases firmness ↓ increases cutting strength	[87]
gluten-free cookies	5, 10 and 15%	↑ flow behavior and viscoelastic parameters ↑ dietary fiber content ↓ increases core hardness (5% optimal value)	[88]
bread	10, 20, 30 and 50%	↑ dietary fiber ↑ total polyphenol content ↓ water absorption capacity is unstable at 50% addition ↓ decrease in hardness and shear strength (all variants are optimal depending on the desired final characteristic)	[89]
Italian salami	7 and 14%	↑ dietary fiber ↑ phenolic compounds ↑ lower calorie content (7% optimal value)	[41]
mayonnaise	2, 4 and 6%	↑ physicochemical, sensory, and rheological parameters ↑ bioactive compounds ↑ texturizing agent (4% optimal value)	[90]
biodegradable food packaging film	8%	↑ total polyphenol content ↑ antioxidant activity	[91]
hydrogel	drying in an oven at 55 °C for 12 h; homogenization and drying in an oven at 55 °C for 12 h; homogenization and freeze-drying; ground and sieved, resulting in three particle sizes (>400 µm, 250–400 µm and <250 µm)	↑ total phenolic content and antioxidant capacity ↑ thickening capacity (oven-dried at 55 °C with particle size 250–400 μm and (particle size >400 μm—optimal variant)	[42]

↑ increased the parameters; ↓ decreased the parameters.

**Table 4:** The effect of adding CPP to certain food composites.

Food Composites	Application Conditions	Results	References
chicken sausages	6%	↑ dietary fiber content ↑ acceptability	[92]
bread	15%	↑ antioxidant properties ↑ long-lasting color ↓ intense aroma	[93]
	5, 10, 15 and 20%	↑ nutritional and sensory value ↓ decrease in specific volume and elasticity (10% optimal value)	[54]
	10, 20 and 30%	↑ specific volume, moisture content; ↑ dietary fiber ↓ fat content ↓ protein ↓ sensory properties ↓ rheological properties (10% optimal value)	[94]
pasta	10, 20 and 30%	↑ dietary fiber, water absorption, swelling index ↑ consumer acceptability (10% optimal value)	[58]
cookies	5, 10, 15, 20 and 25%	↑ weight, thickness, and hardness ↓ elongation factor and diameter (10% of the optimal value)	[95]
	72- and 120-mesh particles in proportions of 10, 15, and 20%	↑ dietary fiber ↑ functional and rheological properties (↑ 120 mesh) ↓ protein	[96]
oatmeal cookies	5% and 10%	↑ elasticity and texture. ↑ polyphenols, fats, and minerals ↓ hardness ↓ color (altered hues) ↓ protein (10% optimal value)	[97]
gluten-free cookies	10, 20 and 30%	↑ viscosity ↑ uniformity index ↑ density and pH ↓ color (30% optimal value)	[98]
milk-based beverages	2, 4 and 6%	↑ dietary fiber, carotenoids, and vitamins ↓ acidity (slightly increased) (4% optimal value)	[99]
meatballs	1, 3 and 4.2%	↑ dietary fiber ↑ sensory properties ↑ functional properties (3% optimal value)	[74]
fish roe salad	6 and 12%	↑ physical and chemical properties ↑ sensory properties (12% optimal value)	[100]
gluten-free pasta	5 and 10%	↑ hydration, cohesion, and cooking parameters ↑ nutritional parameters ↓ color (10% optimal value)	[101]
gluten-free muffins	5 and 10%	↑ dietary fiber ↑ reduces hardness (5% optimal value)	[102]

↑ increased the parameters; ↓ decreased the parameters.

## Data Availability

The original contributions presented in this study are included in the article. Further inquiries can be directed to the corresponding author.

## Abbreviations

The following abbreviations are used in this manuscript:

AOAC	Association of Official Analytical Chemists
AP	Apple pomace
APP	Apple pomace powder
CP	Carrot pomace
CPP	Carrot pomace powder
DNS	The dinitrosalicylic acid method
FAO	Food and Agriculture Organization
HC	Hydrocolloid
HPLC	High-Performance Liquid Chromatography
ICC	International Association for Cereal Chemistry
LCA	Life cycle assessment
LC-HRMS	High-resolution liquid chromatography mass spectrometry analysis
MW-HPCO_2_	Microwave-assisted high-pressure CO_2_ system
OHC	Oil holding capacity
RSM	The response surface methodology
TPC	The phenolic content
WF	Water footprint
WHC	Water holding capacity
XG	Xanthan gum
